# Reviewing Psychological Practices to Enhance the Psychological Resilience Process for Individuals with Chronic Pain: Clinical Implications and Neurocognitive Findings

**DOI:** 10.1007/s11916-025-01373-4

**Published:** 2025-03-22

**Authors:** Elif Çalışkan, Füsun Gökkaya

**Affiliations:** Department of Psychology, Institute of Postgraduate Education, İzmir Tınaztepe University, İzmir, Türkiye

**Keywords:** Chronic pain, Psychological resilience, Psychological practices, Positive adaptation, Pain perception, Emotion regulation

## Abstract

**Purpose of Review:**

Psychological practices have emerged as promising treatments for coping with chronic pain (CP) as a psychological resilience (PR) enhancer mechanism. These practices contain cognitive, behavioral and emotional modulation of pain. In this regard, classical cognitive-behavioral therapy (CBT) and current trends in CBT, including acceptance and commitment therapy and mindfulness-based practices may demonstrate significant improvements in pain perception, physical functioning, catastrophic beliefs and fear-avoidance behaviors among patients with CP. However, understanding the neurocognitive mechanisms of these practices includes challenges, such as the need to identify associated brain regions with PR to CP. Our review explored psychological practices to enhance PR as a dynamic neurocognitive process (e.g., changing affect) rather than only being a static trait.

**Recent Findings:**

Psychological practices have promising results in improving positive outcomes for CP sufferers. To illustrate, along with superior PR scores, higher positive affect, adaptive pain beliefs, and physical functioning were reported after these practices. Conversely, lower pain catastrophizing, pain-related fear-avoidance, and self-reported pain ratings were seen as PR factors. Moreover, enhanced PR process may be associated with increased activity of the brain regions, including prefrontal cortex and orbitofrontal cortex, whereas diminished activity, reactivity, and functional connectivity in the anterior cingulate cortex, amygdala and insula.

**Summary:**

This review discusses the neurocognitive modulation of CP through psychological practices and highlights the role of enhancing the PR process for individuals with CP. As the field continues to evolve, understanding the importance of psychological practices to develop PR-related factors is crucial for increasing pain management outcomes.

## Introduction

Pain is one of the most universal experiences of humans and sometimes persists beyond the usual recovery period. It is usually defined as chronic when it lasts or recurs for more than 3 to 6 months [1, 2]. Chronic pain (CP) might be any kind of pain (e.g. headache, migraine, back pain, musculoskeletal pain, or fibromyalgia) [3]. The review by West et al. noted that CP may lead to anxiety, catastrophizing, depression, fatigue or interfere with undertaking usual chores and activities, thus decreasing living standards and increasing medical expenses of individuals [4]. Since it affects more individuals than cancer, heart disease and diabetes, reviewing good practices for coping with CP is extremely important [5]. Yet, none of the most widely used pharmacological, medical or surgical treatments are effective for relieving pain or significantly boosting people’s physical and emotional functions so it requires addressing psychological factors to cope with CP experience [6]. CP has a significant negative impact on brain areas involved in cognitive and emotional modulation of pain [7]. However, regulating emotions and attention by promoting active efforts such as practicing reappraisal, acceptance, distancing or imagery strategies may strongly modulate pain perception (PP) [8]. Moreover, applying emotion and attention regulation strategies may be associated with an enhanced psychological resilience (PR) process, which is an important concept in pain modulation [9].

Psychological resilience (PR) is evaluated as a new paradigm for adaptation to CP. While “higher-order” PR resources are evaluated as stable positive characteristics such as extroversion or optimism, “state” resilience factors can provide a more evolving form of PR on the daily adaptation to CP, such as daily changes in positive affect or favorable social interactions [10]. In fact, PR may shape how people perceive their pain and react to the pain episodes, thus influencing the pain affect (e.g., unpleasantness) and therapeutic outcomes [11]. However, not all individuals who encounter adverse experiences, like pain show positive adaptation, Therefore, there is a great interest in understanding what makes some individuals with CP resilient and how PR is increased [5, 9]. While genetic factors have a significant role in building PR, cognition–emotion–perception processes regulate in coping with adversity. A cognitively based evaluation of an adverse experience shapes an individual’s positive adaptation. Since the pain experience is an unexpected life event, it may increase individuals’ awareness of physical PP which leads to maladaptive behaviors (e.g., avoidance of activities) [9]. However, psychological practices, such as varied forms of cognitive behavioral therapies (CBT) may enhance the PR process for the positive adaptation to living with CP [4, 5, 12].

This review aims to critically examine psychological practices with a particular focus on CBT-based perspective to enhance the PR process for those with CP, suggesting that PR is a dynamic neurocognitive process rather than only being a static trait. This article might be substantial to compile neurocognitive aspects of pain management as a psychotherapeutic intervention. Along with classical CBT, we cover current trends in CBT, including acceptance and commitment therapy (ACT) and mindfulness-based strategies. Thus, both chronic pain sufferers and mental health professionals can utilize this review as a prominent resource to alleviate subjective physical pain and its negative impacts on patients’ functional status. Certain strategies which are activated endogenously by cognitive, behavioral and emotional factors may decrease afferent nociceptive signals to the brain and activate descending pain modulatory system (DPMS) as a natural pain killing mechanism [7]. As discussed in the next section, we review PR as a dynamic process for positive CP adaptation. Thereafter, we cover cardinal psychological practices to enhance the PR process for coping with CP. Later, we explain limitations and future directions. Lastly, we conclude the paper by highlighting the importance of neurocognitive modulation of pain in the PR process.

## Psychological Resilience: A Dynamic Process for the Positive Adaptation to Chronic Pain Experience

PR is defined as a dynamic process which is associated with both stable individual characteristics (resilience resources) and state factors (resilience mechanisms) to bounce back and adapt to adverse outcomes over time [13]. Therefore, it is best conceptualized PR as a dynamic process determined not only by the individual characteristics, but also by psychological factors that are presented to the advantage of the person for successful adaptation to living with CP [10]. From this perspective, it is possible for anyone to be taught to engage in using different protective factors that may be available to them [13, 14]. For instance, while a particular activity might be amusing and valuable for one’s daily life, it can aggravate pain after a certain time, however, a person can show a positive adaptation to his pain by using adaptive strategies, such as cognitive reappraisal (i.e., changing how one interprets the meaning of his emotions or thoughts about aversive stimuli) to think the pain experience as an opportunity to find new activities that will be less likely to cause pain (e.g., shifting from cycling to walking) instead of interpreting it as a catastrophic even [15]. Due to the dynamic neurocognitive nature of PR, it might be associated with various cognitive-behavioral mechanisms, including adaptive emotion regulation (ER) strategies and certain brain regions [16, 17]. Recent developments in CBT, including mindfulness and ACT interventions were found effective for CP sufferers [6, 12, 18, 19].

PP is often shaped by somatosensory information resulting from actual threat to the body, it may be also affected by emotions, beliefs, expectations or attention [8]. According to the gate control theory of pain, the spinal cord is the first main meeting point for the nervous system to drive the sensory experiences and behavioral responses. There is a gate mechanism for pain modulation located in the dorsal horn of the spinal cord [20]. To perceive a sensation as pain, pain signals need to be passed through to the brain from the gates. When the gates are more open than at other times, pain signals are more likely to go through to the brain and people experience a high level of pain. However, if the gates are more closed, pain signals will be blocked from traveling up to the brain and people feel less pain. The theory suggests that psychological factors, such as emotions, attention or past experiences may influence PP by regulating the gate system [21]. Anxiety, worry or depression can aggravate pain by activating the central control trigger so that the gate is opened, whereas distraction, relaxation or positive emotions can lead to the gate to be closed and it decreases PP [22].

As with PP, PR is a top-down process (initiated with thoughts, knowledge, experience, emotions, or expectations) and bottom-up system (initiated with sensory input or the stimulus) to maintain adaptive projection between the perceived adverse event and the sensory stimulus [9].While ascending sensory pathways work in a bottom-up manner, descending sensory pathways (also called DPMS) work in a top-down way [23]. DPMS is significantly affected by psychological factors and altered activities of neurotransmitters (e.g., serotonin and norepinephrine) in DPMS can be seen in CP conditions [24]. DPMS includes facilitatory and inhibitory pain pathways. In other words, descending facilitatory pain pathways (DFPP) are responsible for enhancing PP, whereas descending inhibitory pain pathways (DIPP) suppress it at the spinal level) [25]. Increased activity in the DFPP and diminished activity in the DIPP within the spinal cord dorsal horn contributes to the development of CP. While pharmacological treatment may restore the balance between the two pathways by decreasing the DFPP and enhancing the DIPP activity, this treatment may be associated with certain characteristic side effects (e.g., nausea, vomiting), tolerance and drug dependence [24]. Conversely, enhancing PR processes can activate DPMS to provide adaptive pain modulation that can influence PP and nociceptive processing in certain brain regions, thus regulating cognitive (e.g., interpreting the meaning of pain) and emotional factors (e.g., inducing positive emotion despite pain) [25, 26].

The fear-avoidance models describe how some individuals who experience acute pain may become trapped into a vicious circle of chronic suffering. It suggests that patients who interpret pain as non-harmful can confront their fears and continue with engaging daily activities despite pain, which in turn leads to a reduction in fear and recovery in pain over time, whereas patients who interpret pain as a catastrophic threat go into a vicious circle that leads to fear, which in turn influences defensive responses, such as avoidance behaviors and hypervigilance (a state of heightened awareness to sense of pain) [27, 28]. An excessive irrational fear of movements or physical activities is sometimes termed ‘‘kinesiophobia’’ [29] Gracely et al. showed positive relationship between catastrophizing scores and increased activity in brain areas related to attention to pain (dorsal anterior cingulate cortex (ACC), dorsolateral prefrontal cortex (PFC), anticipation of pain (medial frontal cortex (mPFC), cerebellum), emotional aspects of pain (claustrum, closely connected to amygdala) and motor control [30]. ACC and insula are the brain regions particularly related to pain unpleasantness and encoding emotional and attentional aspects of pain [7]. The review by Sturgeon et al. indicated that diminished activity, reactivity, and functional connectivity in amygdala (fear network in the brain), ACC and insula may be related to greater PR, whereas high PFC activity may be associated with superior PR [31].

Since the brain regions involved in pain processing are also crucial for emotion and attention, PP may be significantly affected by emotional states and attentional factors [7]. Pain leads to a potent reflex sensory response accompanied by a rapid autonomic and delayed neuroendocrine responses mediated by the sympathoadrenal and hypothalamo-pituitary-adrenal (HPA) axis in that order [32]. Likewise, the sympathetic nervous system (SNS), HPA axis and the dopaminergic and serotonergic neurotransmitter systems are associated with the neurobiology of PR and responsible for managing the stress responses [33]. When the human body experiences a perceived harmful event, it signals the brain to prepare for fight or flight responses. Likewise, in response to pain, increased activity occurs in the SNS and it triggers physiological hyperarousal. Therefore, structural changes can be seen in key regions of the brain in chronic pain experience and the brain regions that process sensory and cognitive-emotional aspects may lead to maladaptive neurobiological alterations in pain processing pathways which increases pain sensitivity and decreases modulation of pain [34].

Although adverse experiences may disrupt brain networks (e.g., mPFC function) and leads to maladaptive cognitive ER processes to adapt to stressful situations, including pain [35, 9], increased gray matter volume in the particular brain regions, such as in PFC or somatosensory cortex (SSC) may contribute to enhancing PR and reducing pain catastrophizing in those with CP [31]. The review stated that mPFC is one of the key regions of PP and can regulate cognitive appraisal to promote PR as a protective factor in the face of adversities [9]. Nonetheless, the human brain has a time-varying malleability and ability to change so it can evolve its structure to adapt to adverse life experiences. This adaptation ability is usually described as plasticity and individuals with higher PR can show more positive adaptations to adversities [36]. Moreover, Hanssen et al. noted the role of positive affect as a PR factor in CP experience, which influences nervous, endocrine, and immune responses for the adaptation to persistent pain [34]. For instance, the orbitofrontal cortex (OFC) is responsible for emotion-guided behaviors and researchers found that boosting positive mood and attitude changes toward pain was associated with increased OFC activity [37].

As a result, adaptive ER strategies, particularly reappraisal and acceptance may relieve negative emotions as a booster in the PR process for the modulation of pain [17, 31]. Pain acceptance is one of the ER strategies that is strongly associated with PR [38]. It refers to acknowledging pain by turning one’s focus towards functional goals and engagement in daily life activities (e.g., joining a meeting despite pain), thus promoting better adaptation to CP [39]. Reappraisal of pain was also found related to PR [40]. Goldin et al. revealed that reappraisal created more brain activation in various brain regions (e.g., dorsomedial PFC), lesser brain responses in amygdala and higher heart rate than acceptance, suggesting that reappraisal can be more effective in down-regulating negative emotion and more effortful than acceptance [41]. McRae et al. showed that reappraisal led to an enhanced activation in mPFC and anterior temporal regions which are associated with processing of affective meaning, whereas distraction (i.e., focusing the mind on something other than negative affect or pain) led to a more significant decrease in amygdala activation than reappraisal activation in PFC and parietal regions which is associated with selective attention [42]. However, Haspert found a negative relationship between distraction and PR to CP [17].

Thus, technological developments in neuroimaging and in measuring the biopsychological aspects of CP provides prominent information regarding the neurobiology of PR and PP processes in CP sufferers. Neurocognitive results showed that ER plays a significant role in PR process and cognitive-behavioral modulation of pain [9]. Although going through adverse experiences may sometimes impair brain networks, certain individuals are more resilient to cope with stressful situations [34]. However, thanks to the neural plasticity of the brain, each individual has a unique ability to alter their neurocognitive structure and function which may lead to improvements in their PR process [36]. In response to this ability to change, clinicians have developed empirically supported psychological practices for patients with CP [12, 43, 44]. In the next part of our review, we will cover these practices for adults with CP problems, while bringing up CP modulation strategies and any element of PR to successfully adapt to living with CP.

## Interventions to Foster Psychological Resilience Process for Patients with Chronic Pain

PR factors, such as positive affect, adaptive pain beliefs or pain acceptance can relieve pain-related distress and improve psychosocial and physical functioning [45]. A systematic review conducted by Wainwright et al. operationalized PR as self-efficacy, active coping, positive affect, positive growth, positive reinforcement, optimism, purpose in life, and acceptance in order to examine the impacts of PR interventions on promoting return to work for those with CP [46]. They noted that most interventions were not effective in enhancing work outcomes or key PR factors but some raised health-related quality of life. Researchers found that slower return to work was associated with higher pain-avoidance beliefs [47, 48]. On the contrary, higher PR was related to lower avoidance of pain and greater pain acceptance [38, 39]. Therefore, the fear of pain is one of the emotions that is identified as crucial in the pain experience which may lead to avoidance of activities, such as work or physical activities [28]. Although feeling fear of pain and showing safety behaviors after injury are vital responses for patients to protect themselves, avoidance from daily activities or certain movements may lead to functional disabilities and extreme guarding behaviors [43, 50]. However, researchers show PR as a protective pathway from functional impairment to fear-avoidance in CP [38, 49, 51].

Moreover, avoidance-based coping makes individuals more vulnerable to maladaptive responses to pain while approach-focused coping contributes to the PR process in pain experience [52]. Kranz et al. found that individuals who show pain willingness (a component of pain acceptance with an open attitude to experience pain without using unnecessary avoidance and control) mainly reduced their negative affect and individuals with greater levels of activity engagement (a component of pain acceptance with an open behavior for pursuing activities) are also better in boosting their positive emotions despite their pain [53]. Exercise therapy with special focus on perceptions and cognitions (e.g., reframing pain memories and imagination of certain feared movements) may play a significant role in retraining the brain about fears and maladaptive perceptions [54]. Dance/movement therapy (DMT) that promotes psychotherapeutic use of movement may also build PR for those with CP by improving patients’ sense of control, creating mind-body connection, connecting to others, reframing and raising emotional wellbeing [55]. Shim et al. found that DMT provided significant improvements not only in PR but also in kinesiophobia, body awareness and pain intensity [56]. Therefore, interventions that promote positive affect may contribute to the PR process in pain modulation and self-reported pain ratings [7, 57], thus decreasing pain sensitivity and increasing well-being [34].

Lastly, increasing positive psychological resources (e.g., hope, optimism) may also foster PR for those with CP [52, 58]. For instance, *the best possible self exercis*e is one of the empirically-supported interventions to induce optimism. In this technique, patients with CP are instructed to imagine and write about their best possible selves about the future. This manipulation led to lower pain intensity and decreased situational pain catastrophizing [59]. The self-directed technique of written emotional description (therapeutic writing) might also increase emotional awareness, while helping individuals to express their positive feelings in language [60]. Individuals with continuous positive and reliable social engagements may have higher resilient outcomes for the successful adaptation to pain [10]. Self-help interventions (e.g., books, digital platforms) allied with therapist support may also foster PR factors, including acceptance, commitment to values, quality of life and life satisfaction for those with CP [61]. CBT combined with adaptive cognitive ER strategies were found efficient in boosting PR, mindfulness and quality of life of CP patients [62]. Both traditional CBT and third wave CBT, such as ACT may play a crucial role in CP experience to enhance PR process [12, 63, 64, 65].

### Overview of CBT in Chronic Pain Experience

CBT is a prevalent treatment, alone or together with medical or interdisciplinary treatments, for individuals with all types of CP problems [66]. CBT was found effective in enhancing PR in patients with CP [62, 63]. Traditional CBT aims to decrease patients’ maladaptive thoughts and behaviors (e.g., catastrophic beliefs, avoidance of daily activities, limping), while increasing adaptive beliefs and physical functioning (e.g., finding new activities or to re-join in pleasurable activities if possible) [18, 66]. Strengths-based CBT suggests that individuals are generally unaware of their strengths and do not see themselves as resilient but they are already resilient in the areas of their interests, committed actions or pleasurable activities since the more they sustain an activity over time, the more they will build PR in the face of adversities. Thus, therapists should look for “hidden strengths” in patients’ everyday lives and raise their strengths into their awareness [67]. CBT includes a variety of exercises, such as encouraging walking programs to increase engagement to meaningful activities but certain physical movements or activities can lead to an increase in pain severity. Therefore, CBT therapists encourage individuals with CP to find new ways of doing enjoyable activities that will be less likely to cause pain [68].

However, experimental studies revealed that individuals with CP, particularly musculoskeletal pain may acquire fear between neutral movements and pain by associative learning. The movements (conditional stimulus) previously paired with pain (unconditional stimulus) may signal danger and hence start eliciting defensive conditional fear responses, such as avoidance behaviors compared to similar movements that were never followed by pain [27]. The fear-avoidance may spread even beyond movements or activities that were associated with pain during the initial pain episode. When a movement predicts pain, pain-related fear may spread selectively to novel movements related to the original painful movement, and not to those similar with original non-painful movement. Such a generalization of fear may lead to increasing risk to respond to false threat alarms, which may trigger persistent fear and avoidance behaviors in CP, while bearing to missing positive threat alarms [29]. Seminowicz et al. showed that CBT in CP may change the perception of noxious signals and decrease pain catastrophizing, while raising gray matter in prefrontal and somatosensory brain regions, as well as pregenual ACC and posterior parietal cortex [44]. This finding may indicate improved cognitive reappraisal of pain, an adaptive ER strategy which may foster PR process [9].

*Exposure in vivo* was found to be a highly effective treatment to reduce pain related fear-avoidance, catastrophizing, and disability [50, 69, 70]. It is one of the CBT techniques particularly for phobias and originally mentioned as graded exposure and it was adapted to target avoidance behaviors of patients with pain-related fear [71]. It involves gradual confrontation with everyday activities or movements in which individuals have catastrophic expectations and avoid doing them [50]. Studies revealed that despite the exposure to activities during the intervention period, pain didn’t increase, rather it decreased [43, 50]. Therefore, engaging in the feared activity or movement until individuals become convinced that the movement can be done without pain or anxiety may contribute to the PR process [69]. Individuals with more frequent pain appraisals as a challenge, greater pain self-efficacy, lower pain catastrophizing and less pain-related dysfunction were associated with higher PR scores [40]. However, it is important to consider that decline in fear-avoidance may not always lead to decrease in pain or disability [69].

Moreover, CBT in CP involves *relaxation training* to decrease stress and muscle tension by using deep breathing exercises, progressive muscle relaxation, meditation and visualization. Relaxation training aims to decrease the activity of the sympathetic nervous system while coping with stress and anxiety [68, 72]. Attention management strategies (e.g., distracting attention by focusing on breath) can be used in therapy for diverting attention from pain [73]. Pain-related stimuli may enhance the perceived severity of pain sensation since experiencing a negative emotion may increase the perceived unpleasantness of the pain and attentional bias toward aversive stimuli. Thus, both attentional and ER mechanisms may have an important role in modulating PP [7] and fostering PR process [9]. By using real-time functional MRI (rtfMRI) to guide pain relieving training, subjects were given several cognitive strategies that they could use to manipulate activity in the rostral ACC, a region presumably involved in PP and regulation. After the training, they reported alleviation in their ongoing level of CP [74].

Consequently, combining CBT with adaptive cognitive ER strategies may enhance PR to CP [62]. Moreover, CBT is an effective treatment not only for relieving subjective pain experience but also to increase pain-evoked neural activation, including dorsolateral and ventrolateral PFC regions that are responsible for modulating pain [75]. Researchers noted that the pain-evoked activity in the S1, insula and ACC, is stronger when a person focuses on pain compared to when he is distracted from pain. However, when people deliberately direct their attention to or away from a painful stimulus, attention-related changes in pain-evoked activity in the insula are associated with activity in the superior parietal cortex, the region that gives the attentional biasing signal for the attentional modulation of pain [7]. Paying attention to negative emotional stimuli has been associated with maladaptive psychophysiological responses to stressful events. Therefore, enhancing attentional bias towards positive emotional stimuli may be a protective resource that contributes to the PR process [76].

### Examining ACT and Mindfulness-Based Practices in Chronic Pain Experience

Clinicians have developed three approaches or “waves” of CBT. While the first two waves have a prevalent attention on either cognitive therapy or behavior therapy, it is somewhat hard to identify the “third wave”. Although the term “third wave” is not used so often in CP management, the developments in CBT provided wider treatment options in coping with CP experience [77]. Third-wave CBT approaches, particularly mindfulness and ACT have been predominant clinical practices in CP modulation [62, 78, 79]. On the one hand, mindfulness aims to be aware of and focus on the present moment while accepting and acknowledging it, without preoccupying with negative thoughts or feelings [80]. On the other hand, the main goal of ACT is to focus on the present moment and accepting thoughts and feelings with *psychological flexibility* (an ability to act in accordance with values and committed actions in the presence of inner discontent) to strongly pursue one’s long-term goals despite obstacles [81].

Researchers showed the importance of psychological flexibility as a PR enhancing mechanism among individuals with CP and anxiety [64]. However, lack of flexibility may lead to avoiding particular situations related to pain and distress in CP experience. Individuals who are excessively controlled by their unpleasant thoughts and feelings may have difficulty in being in the present and engaging in personally meaningful activities. This attitude to eliminate the aversive experience is negatively reinforced and is defined as *destructive experiential avoidance* or *psychological inflexibility* [82]. Conversely, psychological flexibility includes the processes of accepting pain, mindfulness (a process that covers acceptance connecting with the present moment), noticing thoughts rather than caught up into them, and understanding that experiences, thoughts and emotions have ever-changing content [83]. The review by Esteve and Ramírez-Maestre stated that the concepts of PR and acceptance are interconnected and resilient people are more likely to perform an accepting attitude in life and will possibly develop accepting behavior toward CP [84].

Studies revealed that acceptance increases pain willingness and pain tolerance compared to distraction or control strategies [85]. Acceptance is a promising strategy as part of the ACT and mindfulness practices, while reappraisal and distraction are more CBT oriented strategies [17]. Hatami et al. found that both CBT and ACT have fundamental effects on PR in patients with CP [62]. While the former focuses on working toward behavioral goals and problem solving (e.g., increasing behavioral activation), along with activity pacing, relaxation training and cognitive restructuring [66], the latter often uses experiential strategies such as metaphors, exercises and paradoxes in order to weaken the literal language functions and feel more real and tangible in an accepting way [81]. For instance, in the metaphor of *rope pulling*, it is assumed that people pull a rope against their inner discontents and it is encouraged to leave the rope and go on their way together with the discontents that force them. Esmaeili et al. found that ACT enhanced PR and decreased perfectionism in migraine patients while encouraging them to cope with avoided situations and increasing committed actions [65].

Although it is advisable to use mindfulness exercises in ACT, it is not compulsory. Formal mindfulness is evaluated as intentional and non-judgmental awareness which contains Mindfulness-based Stress Reduction (MBSR) and Mindfulness-based Cognitive Therapy (MBCT) [6]. On the one hand, MBSR is a group intervention that promises to help with the treatment of CP and the decrease in physical functioning and psychological well-being due to pain experience. It consists of a variety of techniques including *body scan*, in which patients scan each part of their body for pain, pressure or tension; *awareness of breathing and emotions*, which entails paying attention to breathing and accepting emotions as they happen; *mindful yoga*, which includes to paying attention to movements of the body, and *mindfulness in daily life* to bring awareness to walking, eating, or listening [19]. On the other hand, MBCT is an evidence-based pain treatment that integrates both MBSR activities (e.g., body scan) and classical CBT techniques (e.g., activity planning) [78]. Mindfulness-based CP management provided moderate to large effects not only in PR but also in pain management, acceptance, mental-health-related quality of life, psychopathological symptoms, use of analgesics, self-blame and negative thoughts in general [86].

Thus, the cognitive, behavioral and emotional changes which contribute to the positive adaptation to CP may enhance the PR process [5, 9]. Zeidan et al. showed that mindfulness practices raised pain threshold in comparison to before the training and the control group [78]. Pain relief after brief mindfulness meditation training was associated with activation of the bilateral OFC, rostral ACC, and greater thalamic deactivation, revealing cognitive reappraisal and detached pain-related thoughts, while the pain relief after extensive training was associated with deactivation of prefrontal and greater activation of SSC areas indicating decreased appraisals regarding the sensory aspects of pain and pain-related affect [37]. However, researchers showed that ACT led to higher effects on depression and anxiety than MBSR and MBCT for patients with CP [6]. Aytur et al. found that after the 4-week ACT intervention, brain activation within and between main networks including self-reflection, maladaptive emotions, and cognitive control reduced in patients with musculoskeletal pain, indicating diminished depression and pain interference as well as enhanced involvement in social roles, which may play a key role in the PR process [87].

## Limitations and Future Directions in the Existing Literature

Foremostly, narrative review consists of some limitations, including the missing quality check, and the lacking conclusions for effectiveness [88]. The absence of objective and comprehensive selection criteria may result in distinct bias in the data search method and study findings [89]. There is a similar limitation in this study. This limitation is also related to the nature of research examining the relationship between PR and CP experience. Since PR in CP experience is a new paradigm, conceptual and methodological issues have emerged in defining PR to CP [10]. Definitions of PR to CP have largely been based on self-reported measures and particularly examined by the positive psychology field [31]. Therefore, we are unable to systematically review the psychological practices to enhance the PR process in CP. Additionally, few studies directly examine changes in PR scores after psychological practices in those with CP. Studies that investigate whether CBT-oriented interventions (particularly, classical CBT) increase PR to CP are limited in the literature. In this way, along with PR scores, we also review secondary outcomes (e.g., levels of pain, pain sensitivity, attention to pain, fear-avoidance, catastrophizing, acceptance, reappraisal, positive affect) which are thought to improve PR process. Someway, the covered resources or mechanisms may not always be related to rising PR scores. To illustrate, acceptance has been considered as an interrelating factor with PR [84], whereas Haspert didn’t find any relationship between PR scores and pain regulation with acceptance [17]. Therefore, future studies may particularly investigate the role of CBT-based practices on self-reported PR ratings.

Moreover, PR is a developing concept encompassing positive adaptation to the CP experience [46, 52]. To effectively illustrate PR as a mutable and fluid process, we address cognitive, behavioral, emotional, and neurobiological views. Since few neuroimaging studies have investigated the PR process in individuals with CP [31], this review includes some of the brain regions (e.g., ACC, PFC) that may be more associated with the PR process in the modulation of pain. The neurocognitive domain of PR is a growing area so we couldn’t clarify whether the brain activity that was detected beforehand would predict that somebody may show a particular response to CP. However, future studies may inquire about whether certain neural patterns predict the PR process in CP experience and how we can foster adaptive coping skills to enhance the PR process for individuals with CP. We believe that addressing neurocognitive mechanisms may increase our knowledge of the dynamic PR process for promoting pain relief and successful adaptation to the CP experience.

## Conclusion

In conclusion, instead of evaluating PR only as a trait or mechanism, we review it as a dynamic process that consists of neurocognitive factors. These factors might be associated with how individuals perceive their pain to enhance the PR process in the CP experience. Psychological practices, including traditional CBT, ACT and mindfulness-based interventions can contribute to the enhancement of the PR process (both psychological resources and mechanisms) for promoting positive adaptation to living with CP. However, structural changes can be seen in the fundamental regions of the brain and these changes may be related to the PR process for those with CP (e.g., mPFC, OFC). Maladaptive biological alterations in pain processing pathways may lead to impairment in DPMS. Yet, the human brain has a strong ability to evolve its structure to adapt to adversities. Due to the dynamic nature of PR, it may be associated with the functions of multiple brain regions related to how individuals perceive their pain and respond to it [16]. Therefore, the practices that aim to enhance the aspects of the PR process for individuals with CP (e.g., adaptive ER strategies) may target at activating varied neurocognitive mechanisms associated with PP, thus improving adaptive responses and positive outcomes in the progress of time as natural pain relievers.

## Key References

1. Sturgeon JA, Zubieta C, Kaplan CM, Pierce J, Arewasikporn A, Slepian PM, Trost Z. Broadening the Scope of resilience in chronic pain: Methods, social context, and development. Current Rheumatology Reports, 2024;1–12. 10.1007/s11926-024-01133-0.
  This paper helps to review the neurocognitive mechanisms of the dynamic PR process for the modulation of CP.
  This paper is important to provide limitations and recent developments for the scope of the PR process.

2. Shirvani F, Aliakbari M, Alipoor A, Rafiepoor A. Comparison of the cognitive-behavioral therapy and acceptance and commitment therapy on resilience and diagnostic factors in patients with chronic pain. International Journal of Applied Behavioral Sciences, 2021;8(1):21–33.
  This paper helps to show that PR is a mutable process, instead of a static trait, which can be increased by CBT and ACT-based practices.
  This paper is important to demonstrate that CBT and ACT have the necessary effects on improving PR scores for patients with CP.

3. Haspert V. Improving acute pain management with emotion regulation strategies: A comparison of acceptance, distraction, and reappraisal [Unpublished doctoral dissertation]. University of Würzburg. 2023. 10.25972/OPUS-29866.
  This paper helps to provide an overview of ER strategies, including acceptance, distraction and reappraisal to relieve negative emotions as a PR factor.
  This paper is important to show that ER strategies, particularly reappraisal and acceptance may increase modulation of pain and activate certain brain regions as a PR mechanism to CP.

## Data Availability

No datasets were generated or analyzed during the current study.
